# Prompts to Disrupt Sitting Time and Increase Physical Activity at Work, 2011–2012

**DOI:** 10.5888/pcd11.130318

**Published:** 2014-05-01

**Authors:** Ann M. Swartz, Aubrianne E. Rote, Whitney A. Welch, Hotaka Maeda, Teresa L. Hart, Young Ik Cho, Scott J. Strath

**Affiliations:** Author Affiliations: Aubrianne E. Rote, University of North Carolina–Asheville, Asheville, North Carolina; Whitney A. Welch, Hotaka Maeda, Young Ik Cho, Scott J. Strath, University of Wisconsin–Milwaukee, Milwaukee; Teresa L. Hart, University of Wisconsin–Milwaukee, Milwaukee, Wisconsin, and Arizona State University, Phoenix, Arizona.

## Abstract

**Introduction:**

The objective of this study was to assess change in sitting and physical activity behavior in response to a workplace intervention to disrupt prolonged sitting time.

**Methods:**

Sixty office workers were randomized to either a Stand group (n = 29), which received hourly prompts (computer-based and wrist-worn) to stand up, or a Step group (n = 31), which received the same hourly prompts and an additional prompt to walk 100 steps or more upon standing. An ActivPAL monitor was used to assess sitting and physical activity behavior on the same 3 consecutive workdays during baseline and intervention periods. Mixed-effect models with random intercepts and random slopes for time were performed to assess change between groups and across time.

**Results:**

Both groups significantly reduced duration of average sitting bouts (Stand group, by 16%; Step group, by 19%) and the number of sitting bouts of 60 minutes or more (Step group, by 36%; Stand group, by 54%). The Stand group significantly reduced total sitting time (by 6.6%), duration of the longest sitting bout (by 29%), and number of sitting bouts of 30 minutes or more (by 13%) and increased the number of sit-to-stand transitions (by 15%) and standing time (by 23%). Stepping time significantly increased in the Stand (by 14%) and Step (by 29%) groups, but only the Step group significantly increased (by 35%) the number of steps per workday. Differences in changes from baseline to intervention between groups were not significant for any outcome.

**Conclusion:**

Interventions that focus on disrupting sitting time only in the workplace may result in less sitting. When sitting time disruptions are paired with a physical activity prompt, people may be more likely to increase their workday physical activity, but the effect on sitting time may be attenuated.

## Introduction

Since the middle of the 20th century, social, cultural, environmental, and technological influences have encouraged sedentary behavior in the occupational domain. These influences have resulted in an increased number of sedentary and light-intensity occupations and a reduction of 124 kcals/day to 140 kcals/day in occupation-related energy expenditure (1,2). Office workers spend 65% to 75% of their workday sitting, with 33% to 64% of sitting time in bouts of 20 minutes or more or 22% to 60% in bouts of 30 minutes or more, depending on how sitting time is assessed (3–5). Adults spend 50% to 68% of waking hours (7.3–9.3 h/d) being sedentary, defined as “any waking activity characterized by an energy expenditure ≤ 1.5 metabolic equivalents and a sitting or reclining posture” (6–8). Large amounts of daily sedentary behavior can negatively affect health and longevity and therefore warrants attention (9–11).

Worksite wellness programs are common. Companies are interested in improving and maintaining employee health to reduce health care costs, improve worker satisfaction, improve productivity, and reduce sick time. Most of these programs focus on increasing physical activity. Recently, research has focused on decreasing total sitting time and breaking up prolonged sitting at work through education (4,5), computer software (3), or sit–stand workstations (12–14). Although interventions have succeeded in changing sedentary behavior or physical activity independently, interventions have yet to combine recommendations to change both sedentary and physical activity behavior simultaneously. Simultaneously targeting both health-related behaviors in a worksite intervention may produce better health outcomes.

The objective of this study was to assess change in sitting and physical activity behavior in response to a workplace intervention to disrupt prolonged sitting time. We hypothesized that pairing recommendations for changing sedentary behavior and physical activity would result in less sedentary behavior and more physical activity.

## Methods

### Participants

Full-time employees (employed ≥20 y) engaged in a sedentary occupation were recruited to complete this intervention from September 2011 through December 2012. Recruitment consisted of identifying employees with clerical positions at a large, Midwestern American university through the university directory. Participants were included if they sat for more than 60% of their workday, as measured through verbal self-report. Exclusion criteria included use of an assistive device such as a cane or walker, gait impairment, a fracture of a lower extremity within the previous 3 months, or an amputation of any part of the lower limb other than toes. The study protocol was approved by the university institutional review board. Informed consent was provided by all participants.

### Protocol

This intervention was a parallel-group, randomized trial that consisted of 3 meetings (1 in a laboratory and 2 at each participant’s workstation) and 2 periods of measurement (baseline and intervention). During the first visit, participants reported to the laboratory, where they provided consent and completed a health history questionnaire; researchers measured height and body mass following standardized procedures with a Detecto 339 stadiometer and balance-beam scale (Detecto, Webb City, Missouri) (15). Participants were then given instructions on how to wear the activPAL motion and postural assessment monitor (PAL Technologies, Glasgow, United Kingdom) during the baseline and intervention periods. During all working hours for the same 3 consecutive workdays during 2 successive weeks (baseline and intervention weeks), all participants wore the activPAL and a wrist watch (that prompted participants with beeps or vibrations); the Step group also wore a pedometer. The baseline period began at least 1 day (but no more than 5 days) after the laboratory visit. During the 3-day baseline monitoring period, participants were asked to put the monitor on immediately on arrival at the office, not to change their usual behavior, and remove the monitor just before going home. Participants were also asked to record in a paper log the exact times they put on and took off the monitor. After the baseline monitoring period, research staff met each participant at his or her workstation and collected the monitor. 

Random number generation was used to assign participants to either the Stand group or Step group. Assignments were written out and placed in sealed, numbered envelopes. The envelopes were opened sequentially by a researcher, participants were informed of group assignment, and a new monitor was issued to each participant. The 3-day intervention monitoring period began on the same day of the week as the baseline monitoring period, during the week following baseline. During the intervention period, participants were asked to follow the intervention protocol, which included the same monitor procedures as the baseline period.

### Intervention

The intervention was designed to disrupt 60 continuous minutes of sedentary behavior. Previous research recommended a 5-minute break every hour (16), and another study suggested that office workers can achieve this goal (5). Participants were randomized into one of 2 intervention groups (Figure), and participants in both groups received a prompt from a wrist-worn device and a desktop computer application to disrupt their sedentary time. The wrist-worn prompt was set to beep (Armitron MD0346-R(T)-2, Armitron, Long Island City, New York) or vibrate (WobL Watch, PottyMD, Knoxville, Tennessee) once per hour; participants selected the type of monitor they wanted to wear. The computer prompt was a free download (TimeLeft, NesterSoft Inc, Woodbridge, Ontario) that provided a pop-up message every hour. The Stand group participants were asked to get up from their chairs when the computer prompt stated, “Hello, please get out of your chair.” Participants were not given further instructions on what to do while upright (how long to stand or other activities). The Step group participants were asked to get up and walk at least 100 steps when they received the computer prompt “Hello, please get up and walk at least 100 steps.” Step group participants were given a pedometer (Yamax SW-200 Digi-Walker Pedometer, Yamasa Tokei Keiki Co, Toyko, Japan) to facilitate this goal.

**Figure:**
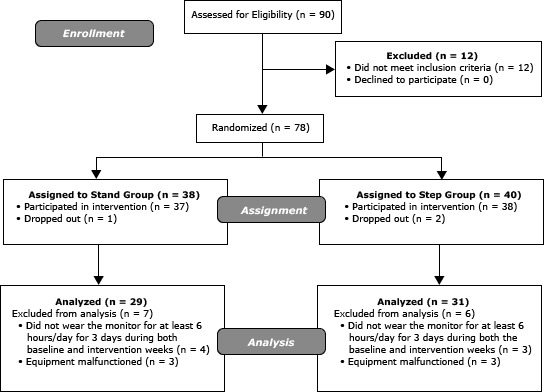
Enrollment, participation, and analyses: intervention to disrupt sitting time and increase physical activity among clerical workers at a Midwestern University, 2011–2012.

### Measures

Sitting, standing, and stepping behaviors were assessed by using the activPAL accelerometry-based motion and postural monitor. The activPAL is a uniaxial piezoresistive accelerometer and inclinometer that is small (35 mm × 53 mm × 7 mm) and lightweight (20 g) and measures both dynamic and static movements. It was attached to the midline of the anterior aspect of the right thigh with a manufacturer-supplied adhesive (PALstickie). The ActivPAL measures the posture of an activity according to the longitudinal axis of the thigh. Data were collected at a predetermined 10 Hz and in 15-second intervals. The ActivPAL is a reliable and valid device for the detection of posture and movements during activities of daily living among both young and old adults (17,18). Further technical details of the ActivPAL can be found elsewhere (18,19).

Recorded output from activPAL monitor was downloaded, processed, and classified into sitting, standing, and walking by using manufacturer-supplied activPAL software (version 5.9.1.1, PAL Technologies, Glasgow, United Kingdom). Further cleaning and processing of event data was performed in R version 3.0.2 (www.R-project.org). Information recorded on a paper log was used to determine the start and end of each workday. Three complete workdays of a minimum of 6 hours were required for inclusion. We defined a workday as approximately 8.2 hours. The primary outcome measures, indicative of sedentary behavior, were total time sitting, average duration of sitting bouts, duration of the longest sitting bout, number of sitting bouts of 30 minutes or 60 minutes or longer, and the number of sit-to-stand transitions. Average duration of sitting events was calculated by summing the duration of all sitting events and dividing by the number of sitting bouts. Steps per day, time standing, and time stepping were secondary outcome measures, representing physical activity in the workplace.

### Statistical analysis

A sample size of 30 per group was chosen a priori to achieve 80% power (2-tailed, *P* < .05) to detect a minimum pre–post change of 30 minutes (standard deviation, 60 minutes) in a 6-hour workday for the primary outcome variable: sitting time (20). Descriptive statistics were calculated for age, body mass, height, and body mass index (BMI). Averages at baseline and postintervention and changes in averages were estimated according to mixed effect models with random intercepts and random slopes for time (baseline and postintervention change), controlling for mean-centered age, sex, and BMI. Analyses were performed with Stata 13 (StataCorp LP, College Station, Texas). Significance was set at an α level of *P* < .05.

## Results

Sixty participants completed the intervention. Participants had an average BMI of 28.5 (standard deviation [SD], 7.4); most (68%) were women; all worked as secretaries or administrators (Table 1). We found no significant differences between the Stand group (n = 29) and Step group (n = 31) for age, body mass, height, or BMI (Table 1). Participants wore the monitor for approximately 8 hours during baseline (Stand, 489 min [standard error {SE}, 51 min]; Step, 496 min [SE, 68 min]) (Table 1) and intervention (Stand, 483 min [SE, 47 min]; Step, 500 min [SE], 66 min]); we found no between- or within-group differences. Overall per workday, participants spent 6.3 hours (376.5 min [Table 2]) sitting, 1.3 hours (75.2 min [Table 3]) standing, and 39.4 minutes stepping (average 3,574 steps/workday [Table 3]), and they averaged 29.4 sit-to-stand transitions (Table 2). At baseline, we found no significant between-group differences in outcomes.

**Table 1:** Characteristics of Participants (n = 60) at Baseline, Study on Prompts to Disrupt Sitting Time and Increase Physical Activity at Work, 2011–2012

Characteristic	All Participants (N = 60)	Stand Group (n = 29)	Step Group (n = 31)
Sex, % female	68	60	75
White, %	89	90	88
Completed college, %	92	87	97
Age, mean (SD), y	44.3 (11.1)	42.3 (11.6)	46.1 (10.5)
Body mass, mean (SD), kg	83.4 (25.0)	86.6 (23.8)	80.4 (26.0)
Height, mean (SD), cm	170.4 (9.7)	171.7 (8.9)	169.2 (1.4)
Body mass index, mean (SD), kg/m^2^	28.5 (7.4)	29.3 (7.3)	27.7 (7.4)
Time monitor worn, mean (SD), (minutes/workday^a^)	492 (60)	489 (51)	496 (68)

a Workday defined as approximately 8.2 hours (492 min).

**Table 2:** Sedentary Behavior of Participants (n = 60), Study on Prompts to Disrupt Sitting Time and Increase Physical Activity at Work, 2011–2012^a^

Behavior	Group^b^	Baseline	Intervention	Change^c^	*P* value^d^
Sitting time, minutes/workday	Stand	380.2 (8.7)	355.2 (8.9)	−25.0 (9.6)	.009
	Step	377.2 (14.6)	366.3 (14.8)	−10.9 (7.8)	.16
	Total	376.5 (7.9)	358.5 (8.0)	−18.0 (6.2)	.004
Average duration of sitting bout, minutes/workday^e^	Stand	14.3 (1.2)	11.9 (1.0)	−2.3 (0.8)	.005
	Step	15.2 (1.4)	12.2 (0.9)	−2.9 (0.9)	.001
	Total	14.7 (0.9)	12.0 (0.6)	−2.6 (0.6)	<.001
Longest sitting bout, minutes/workday	Stand	101.2 (8.2)	72.0 (4.0)	−29.2 (8.2)	<.001
	Step	99.3 (9.5)	80.1 (7.5)	−19.3 (9.9)	.05
	Total	99.4 (6.1)	75.2 (4.0)	−24.2 (6.4)	<.001
Number of sitting bouts ≥30 minutes/workday	Stand	3.8 (0.2)	3.2 (0.2)	−0.5 (0.2)	.005
	Step	3.3 (0.3)	3.2 (0.4)	−0.1 (0.2)	.69
	Total	3.5 (0.2)	3.2 (0.2)	−0.3 (0.2)	.05
Number of sitting bouts ≥60 minutes/workday	Stand	1.1 (0.1)	0.4 (0.1)	−0.6 (0.2)	<.001
	Step	1.1 (0.2)	0.6 (0.1)	−0.4 (0.1)	<.001
	Total	1.1 (0.1)	0.5 (0.1)	−0.5 (0.1)	<.001
Sit-to-stand transitions, events/workday	Stand	28.3 (2.0)	32.4 (1.8)	4.2 (1.2)	.001
	Step	30.4 (2.0)	32.7 (1.9)	2.3 (1.7)	.16
	Total	29.4 (1.3)	32.6 (1.2)	3.2 (1.0)	.002

a Values are mean (robust standard error). Workday defined as approximately 8.2 hours (492 min).

b Those in the Stand group (n = 29) were asked to get up from their chair when they received the hourly prompts; those in the Step group (n = 31) were asked to get up and walk at least 100 steps when they received the prompt.

c Difference between intervention and baseline within each group. Differences in changes between groups were not significant at *P* = .05 for any behavior.

d Determined for change in behavior from baseline to intervention within each group.

e Average sitting bout calculated as number of minutes of sitting time/number of sitting bouts.

**Table 3:** Physical Activity of Participants (n = 60), Study on Prompts to Disrupt Sitting Time and Increase Physical Activity at Work, 2011–2012^a^

Activity	Group^b^	Baseline	Intervention	Change^c^	*P* Value^d^
Standing, minutes/workday	Stand	69.1 (5.0)	85.4 (9.6)	16.2 (7.5)	.03
	Step	78.3 (7.3)	82.5 (9.4)	4.3 (5.9)	.47
	Total	75.2 (4.4)	85.5 (6.5)	10.2 (4.8)	.03
Stepping, minutes/workday	Stand	38.2 (4.0)	43.7 (4.5)	5.5 (2.6)	.04
	Step	41.3 (2.6)	53.2 (4.5)	12.0 (3.8)	.001
	Total	39.4 (2.3)	48.1 (3.2)	8.7 (2.3)	<.001
Physical activity, steps/workday	Stand	3,474 (352)	4,004 (452)	530 (272)	.05
	Step	3,743 (261)	5,036 (418)	1,293 (334)	<.001
	Total	3,574 (213)	4,485 (311)	911 (219)	<.001

a Values are mean (robust standard error). Workday defined as approximately 8.2 hours (492 min).

b Those in the Stand group (n = 29) were asked to get up from their chair when they received the hourly prompts; those in the Step group (n = 31) were asked to get up and walk at least 100 steps when they received the prompt.

c Difference between intervention and baseline within each group. Differences in changes between groups were not significant at *P* = .05 for any behavior.

d Determined for change in behavior from baseline to intervention within each group.

All participants significantly reduced sitting time by an average of 5%, or 18.0 minutes (from 376.5 min to 358.5 min) (Table 2). Stand participants reduced sitting time by 6.6% (from 380.2 min to 355.2 min), whereas the Step participants had no significant change in sitting time (Table 2). The average duration of sitting bouts decreased by 16% (from 14.3 min to 11.9 min) among Stand participants and by 19% (from 15.2 min to 12.2 min) among Step participants. The number of sitting bouts of 60 minutes or more decreased by 54% (from 1.1 bout to 0.4 bouts) among Stand participants and by 36% (from 1.1 bout to 0.6 bouts) among Step participants. Despite the same frequency of prompts to disrupt sitting time, only the Stand group significantly reduced the duration of the longest sitting bout (by 29%, or from 101.2 min to 72.0 min) and the number of sitting bouts of 30 minutes or more (by 13%, or from 3.8 bouts to 3.2 bouts) and increased the number of sit-to-stand transitions (by 15%, from 28.3 events to 32.4 events) (Table 2).

Time standing increased significantly by 23% for the Stand group, but it did not change for the Step group (Table 3). Time stepping significantly increased by 14% in the Stand group and by 29% in the Step group (Table 3). However, only the Step group significantly increased (by 35%) their number of steps per workday (Table 3).

Outcome variables were not significantly different between the Stand and Step group at baseline or intervention. Furthermore, differences in changes from baseline to intervention between groups were not significant for any outcome.

## Discussion

Pairing recommendations for changing sedentary behavior and physical activity improved patterns of sedentary behavior by reducing the average duration of sitting bouts and the number of sitting bouts of 60 minutes or more and by increasing the amount of time stepping. The Stand group significantly decreased work-based sitting time and improved additional aspects of the pattern of sedentary behavior, including decreasing the duration of the longest sitting bout and the number of sitting bouts lasting 30 minutes or longer and increased time standing and the number of sit-to-stand transitions. The intervention that aimed to disrupt sitting time and increase walking (the Step group) significantly increased the number of steps per day but did not alter total sitting time or other aspects of sitting behavior. These results suggest that interventions to disrupt sedentary behavior at work can affect aspects of sedentary and physical activity behavior. However, the focus of the intervention may influence the outcomes. For instance, when sitting time disruptions were paired with a physical activity recommendation, participants were more likely to increase their total workday physical activity.

Patterns of sedentary behavior for all participants at baseline (76% time sitting; 29 sit-to-stand transitions; and a maximum sitting bout of 99 minutes) were similar to patterns in a study that showed office workers spent approximately 66% of their day being sedentary, engaged in 27 sitting events, and had a maximum sitting event of 98 minutes (5). The same study reported that 12% of sitting events lasted at least 30 minutes, and 5% lasted at least 55 minutes(5). Our study showed slightly lower but generally similar results: 12% of sitting bouts lasted 30 minutes or more, and 4% of sitting bouts lasted 60 minutes or more. These data demonstrate the similarity in sitting patterns of office workers and support habit-driven hypotheses of sitting behavior at work (21).

Recently, numerous studies focused on reducing the amount of workplace sitting time (3,5,12–14,21,22). Evans et al compared the use of education with the use of computer-generated point-of-choice prompts to decrease total sitting time and prolonged (>30 min) sitting periods at work in 28 office workers (3). Total sedentary time was not altered, but the pattern of sedentary behavior improved for the point-of choice prompt group, with a decreased number (−0.11 events/h) and duration (−48 min) of sitting events that lasted longer than 30 minutes (3). In contrast, our study showed a significant reduction in workplace sitting time by the Stand group and changes in the pattern of sitting behavior. Discrepancies between our study and the Evans study in the change in total sedentary time could be attributed to differences in the frequency of the prompt — every 30 min in the Evans study and every 60 minutes in ours. Study participants can consciously or unconsciously ignore the prompt, based on their particular work focus and whether they are at a point when a break can be taken. Furthermore, it may be that every 30 minutes is too frequent to take a break (5,21). Together, our data and the Evans data indicate that the addition of prompts to disrupt sedentary behavior is effective at altering aspects of sitting time in the workplace (3).

Recently, interventions have explored how sit–stand desks affect workplace sitting time; they found objectively assessed reductions in sitting time ranging from 36% to 42% (per study) and subjectively reported reductions in sitting time of 224% (12–14). Alkhajah et al showed that a sit–stand workstation resulted in an average 137 fewer minutes (a 42% decrease) of sitting time during an 8-hour workday after 1 week (12). Healy et al implemented an intervention that included sit–stand workstations and individual and organizational intervention strategies and showed similar results — sitting time decreased by an average 122 minutes, and time in prolonged bouts (>30 min) decreased by an average 65 minutes (13). For both studies, most sitting time was replaced with standing (12,13). The changes demonstrated by the Alkhajah and Healy studies were larger than those observed in our study; the differences in changes between those studies and our study are likely attributable to the intervention used and the habitual nature of sitting for desk work. Interventions that include sit–stand desks provide a situational cue to change behavior through the constant presence of the equipment and coworkers who are also engaging in the behaviors (12,13,21), whereas the software prompts in our study only reminded participants to change their behavior once every hour.

Our study showed that both groups significantly increased time stepping, but only the Step group significantly increased the number of steps. The Step group increased stepping time by 12 minutes per workday (a 29% increase) and almost 1,300 steps per workday (a 35% increase) from baseline, whereas the Stand group increased stepping time by 5.5 minutes (a 14% increase) and approximately 530 steps per workday (a 15% increase) from baseline. The increase in number of steps per workday approached significance in the Stand group. The lack of significance could be due to the large variance in number of steps per workday for that group. Additionally, walking pace may have contributed to differences in steps per workday. However, the increases in stepping time and number of steps were proportional in absolute quantity and in change from baseline for both groups.

This study has several limitations. Findings are limited to the population studied and the setting in which the research was conducted. In general, the participants in this study were highly educated, middle-aged, and white; and the intervention was administered on a one-on-one basis (as opposed to simultaneously to a group) in a university setting. The use of prompts was inexpensive and easy to administer; a larger-scale intervention would be possible. Finally, sedentary behavior and physical activity outside the workplace was not assessed; therefore, changes in these behaviors could have occurred outside of work. This study was strengthened by the use of an objective tool to assess time sitting, standing, and stepping. Furthermore, we used passive electronic prompts to ensure all participants were prompted at the same interval. Passive prompting (compared with active prompting) has been shown to increase the likelihood of adherence to an intervention to disrupt sedentary behavior (21). A logical next step in this area of research would be to evaluate total daily physical activity and sedentary behavior in conjunction with this type of workplace intervention. Evaluation of changes in health variables and long-term adherence to disruptions of sedentary behavior are warranted.

Worksite wellness programs are common today, with aims to manage employee health to reduce health care costs, improve worker satisfaction, and improve productivity and reduce sick time. This study showed that a workplace intervention to disrupt sedentary time or to increase walking at work (or both) is efficacious. Interventions focused only on disrupting sitting time in the workplace can result in less sitting time at work. However, when sitting time disruptions are paired with a physical activity recommendation, people appear more likely to increase their total workday physical activity, and the effect on sitting time is attenuated.
